# Advantages and disadvantages of peri-abortion ultrasonography

**DOI:** 10.1530/RAF-25-0190

**Published:** 2026-02-26

**Authors:** John J Reynolds-Wright, Sharon T Cameron

**Affiliations:** ^1^ Centre for Reproductive Health, University of Edinburgh, Edinburgh, UK; ^2^ Chalmers Centre, NHS Lothian, Edinburgh, UK

**Keywords:** women’s health, pregnancy, control of reproduction

## Abstract

**Abstract:**

Ultrasonography remains an important component of abortion care, as it does in early pregnancy in general. We propose that selective use of ultrasonography, in line with World Health Organization recommendations, allows clinicians and services to maximise the advantages of peri-abortion ultrasound while minimising the impact of the disadvantages of the technology. Patient preferences should be prioritised where possible and safe to do so.

**Lay summary:**

Ultrasound scanning before, during, or after an abortion can be helpful to guide patient care. However, using this for all patients in all scenarios can lead to problems with inappropriate diagnosis or over-diagnosis of conditions and complications. We recommend that ultrasound scanning be used only when needed and guided by evidence-based guidelines and protocols, in line with the World Health Organization.

## Introduction

The World Health Organization abortion care clinical guidelines (World Health Organization 2022) recommends against the use of ultrasound as a pre-requisite to accessing abortion. Pre-abortion ultrasound is used universally in many settings, whether due to legal requirements, due to clinical guidelines advocating for this, or to facilitate service delivery. Some settings may also use routine ultrasound as a method of post-abortion assessment to confirm complete abortion. However, universal use of ultrasound restricts the locations from which abortion care can be provided and increases system costs and individual costs, as ultrasound machinery needs to be bought and maintained and staff trained to conduct ultrasonography.

## Selective ultrasonography prior to abortion

While universal pre-abortion ultrasound location and dating of pregnancy is unnecessary, selective use of ultrasonography – based upon the clinical presentation of the patient – can be valuable to guide selection of treatment protocols and identify pathological pregnancy or advanced gestation that may require more specialist treatment. Patients may themselves express a preference for pre-abortion ultrasonography, and this should be facilitated where possible.

Services providing abortion care should develop a mechanism by which to determine the need for ultrasonography. There are several published structured protocols and evidence reviews to support this (Raymond *et al.* 2020, Ralph *et al.* 2022, Upadhyay *et al.* 2022, Pearlman Shapiro *et al.* 2023, Kerestes *et al.* 2024). As a minimum, assessments should include screening questions to determine risk of ectopic pregnancy and advanced gestation; see Table 1.

**Table 1:** Screening questions to support decision to provide pre-abortion ultrasonography.

Domain to consider	Specific questions	Detail
Signs or symptoms of ectopic pregnancy	Are you experiencing abdominal pain?	If yes – pre-abortion ultrasound scan required to confirm the location of the pregnancy
	Have you had bleeding since you became pregnant?	
Major risk factors for ectopic pregnancy	Have you had an ectopic pregnancy before?	
	Have you had surgery to your fallopian tubes, including female sterilisation?	
	Did you have an IUD in place when you became pregnant?	
Is LMP reliable for determining gestation?	Do you know the date of your last period? How confident are you of this date?	If very uncertain or no idea of LMP, scan required
	Are you breastfeeding or have recently stopped?	If currently breastfeeding or stopped within last month, pre-abortion ultrasound indicated, but see below
	Have you recently had another pregnancy? When was this?	If had another pregnancy with a certain date of completion (birth, abortion, miscarriage, etc.), this can help determine current gestation. For example, if gave birth 10 weeks ago, cannot be more pregnant than this, so ultrasound for gestation not required
	Have you used any hormonal medicines, including contraception or emergency contraception, in the last 3 months?	If yes, should prompt ultrasound as LMP may not be reliable
	When did you last have a negative pregnancy test?	Can be useful to aid in determining gestation in patients with infrequent menses
	Are you certain regarding the timing of the sexual contact that made you pregnant?	The patient may have only had sexual intercourse at a particular time in last few weeks, for example, their partner works away for many weeks at a time and the patient has no other partners

LMP, last menstrual period.

Screening should include the following:

- date of last menstrual period – using a patient’s self-reported last menstrual period date, when this is available, has been shown to be accurate when compared to ultrasonographic gestational dating in the first trimester (Olesen *et al.* 2004, Sundermann *et al.* 2025),
- consideration of use of hormonal routine or emergency contraception as this may alter the accuracy of last menstrual period date,
- presence of intrauterine contraception during the pregnancy as this presents an increased risk of ectopic pregnancy (Heinemann *et al.* 2015), and
- other significant risk factors for ectopic pregnancy, such as history of ectopic pregnancy or tubal surgery.

Symptoms of pain or bleeding are also indications for pre-abortion ultrasonography as these might indicate ectopic pregnancy or a failing pregnancy (Royal College of Obstetricians & Gynaecologists 2020, 2022).

Although large observational studies of abortion delivered using selective pre-abortion ultrasonography have reported that this approach is a highly accurate means of confirming gestation, no screening algorithm is perfect and so there will always be some cases of a later than expected gestation, but these cases are rare (approximately 1 in 3,000) (Aiken *et al.* 2021, Reynolds-Wright *et al.* 2021).

## Advantages of routine pre-abortion ultrasonography

The advantage of performing pre-abortion ultrasonography includes accurate determination of gestation, location, and the number of pregnancies (single or multiple). This can be helpful for formulating an appropriate treatment plan, for example providing the correct dosing of misoprostol appropriate to gestation for medical abortion or scheduling the most appropriate form of surgical abortion.

Prior to 10 weeks of gestation, 200 mg mifepristone orally, followed by a single dose of 800 μg misoprostol sublingual/vaginal/buccal, is a highly effective regimen in over 97% of cases (National Institute for Health & Care Excellence 2019). However, with increasing gestation, additional doses of misoprostol 400 μg may be needed to facilitate timely completion of abortion (World Health Organization 2022) and so ultrasonography prior to abortion can be used to provide an accurate gestation and allow for prescription and dispensing of an appropriate amount of misoprostol to maximise success of medical abortion. For surgical abortion, the procedure changes from vacuum aspiration to dilatation and evacuation after 14 weeks (World Health Organization 2022). In many health systems, fewer providers have the skillset to provide surgical abortion beyond 14 weeks (Lohr 2008, Stifani *et al.* 2022, Sium *et al.* 2024) and so accurate gestation is useful for procedure planning and ensuring that patients receive the optimal surgical procedure and anaesthetic choice. While local anaesthesia alone might be sufficient for vacuum aspirations at early gestations, sedation or general anaesthesia is usually required for dilatation and evacuation to be tolerable (World Health Organization 2022) and ultrasound dating can be helpful to plan this.

Pre-abortion ultrasonography may also detect an asymptomatic failed pregnancy (missed miscarriage). In this scenario, women would have the option to choose expectant management (Al-Ma’ani *et al.* 2014), rather than medical management with mifepristone and misoprostol or surgical evacuation. For some women, knowing that the pregnancy has already ended may be important.

Ectopic pregnancies represent approximately 1–2% of all pregnancies, and as women present to abortion services earlier in pregnancy, those ectopic pregnancies may not have yet become symptomatic. However, the value of universal pre-abortion ultrasonography for detecting asymptomatic ectopic pregnancies in low-risk individuals is questionable, as it has been shown that it may aid detection of some cases but may provide false reassurance that a pregnancy is intrauterine in others (Duncan *et al.* 2022).

Another form of pathological pregnancy, the molar pregnancy, may be identified using pre-abortion ultrasonography. Between 0.57 and 2 per 1,000 pregnancies are molar in nature (Lurain 2010). As with ectopic pregnancies, in areas with good access to abortion, these pregnancies may present to abortion services prior to becoming symptomatic. Ultrasound imaging can be helpful to identify these comparatively rare pathological pregnancies and allow for recommendation and planning of surgical evacuation of the uterus and appropriate monitoring (Ngan *et al.* 2021).

Some patients may also wish to know if they are carrying a singleton or multiple pregnancies. When proceeding to medical or surgical abortion, treatment regimens do not change based upon multiple pregnancies, but the procedure duration (both medical and surgical) may be longer with multiple pregnancies compared to singletons (Hayes *et al.* 2011).

Pre-abortion ultrasonography might also identify uterine structural abnormalities relevant to procedure planning, such as large fibroids or a congenital anomaly such as uterus didelphys or bicornuate uterus. For those with one of these congenital uterine anomalies, a medical abortion may be preferable to a surgical abortion to maximise procedural success and avoid the risk of perforation. However, if selecting surgical abortion, this should be done with continuous ultrasound guidance.

For those with large fibroids, information on their size and site may helpful to the clinician performing a surgical abortion.

A finding of uterine cavity distortion (acquired or congenital) on pre-abortion ultrasonography may also be helpful for counselling on post-abortion contraceptive options since intrauterine methods will unlikely be suitable for those with a marked uterine structural abnormality (Gerkowicz *et al.* 2019).

## Disadvantages of routine pre-abortion ultrasonography

In some settings with easy access to ultrasonography, providers may prefer to have universal pre-abortion ultrasonography. While confirmation of accurate gestation using ultrasound may facilitate service provision by non-medical providers or those with less confidence in using screening tools to determine the need for pre-abortion ultrasonography, universal use may conversely result in greater need for medical input to interpret incidental findings and advise on any further course of investigation.

A common incidental finding is a simple corpus luteum cyst. If visualised, then this may result in a decision to arrange further repeat ultrasonography. However, these are physiological and will resolve spontaneously following resolution of the pregnancy (Petousis *et al.* 2022).

Another incidental finding can include an empty uterus without positive signs of an ectopic pregnancy, likely indicating either an early intrauterine pregnancy or a complete abortion, depending on history of pain or bleeding. In this scenario, correlation with clinical findings and serum hCG testing is necessary to indicate which of these pregnancy conditions is most likely, but often repeat ultrasound or serial hCG testing is required to clarify. While there are studies (Brandell *et al.* 2022, 2024) and protocols (Nippita *et al.* 2025) for managing abortion before ultrasound confirmation of pregnancy location, this can lead to multiple visits for patients and, in some settings, the need to ‘prove’ a viable pregnancy before proceeding to abortion, which can feel counterintuitive and upsetting for patients.

It is also possible that structural uterine abnormalities, as outlined in the previous section, may have been misidentified during pregnancy due to distortion of the pelvic organs by the presence of the pregnancy. Further ultrasonography is then warranted following resolution of the pregnancy to evaluate the uterine structure further, which may cause worry and expense to the patient.

There is evidence from a variety of settings that many patients would prefer not to have a pre-abortion ultrasound, as ultrasound is often associated with wanted and continuing pregnancy (Boydell *et al.* 2021, Reynolds-Wright *et al.* 2021, Blaylock *et al.* 2024). Patient preference for or against ultrasonography should be considered and accommodated where it is safe to do so.

### Ultrasonography during surgical abortion

During surgical abortion, ultrasonography can be useful for confirmation of complete evacuation of the uterus and identification of perforation of the myometrium. This is particularly valuable in second trimester surgical abortion (dilatation and evacuation) but can also be used in first trimester vacuum aspiration. Options for ultrasound can include immediate pre- and post-procedure vaginal or abdominal ultrasound, or if an assistant is available, continuous transabdominal ultrasound guidance throughout the procedure. The latter is preferable for dilatation and evacuation procedures and may also improve the speed of completion of procedure as fetal tissue can be visualised for targeted extraction rather than blind removal (Darney & Sweet 1989).

### Ultrasonography after abortion

In older protocols for assessing completion of medical abortion, routine post-abortion ultrasound was used. With the advent of low-sensitivity urine pregnancy testing (which becomes positive over 1,000 mIU/mL hCG), validated following first trimester abortion two weeks after medical abortion treatment (van der Lugt & Drogendijk 1985, Michie & Cameron 2014, Millar & Cameron 2018, Melville *et al.* 2023), the need for ultrasound confirmation of abortion success became less necessary.

Other alternative approaches to post-abortion ultrasonography include multilevel pregnancy tests, which have several test windows that react at a series of hCG concentration thresholds, and can be used to test pre- and post-abortion to demonstrate a reduction in the hCG level of a particular magnitude, thus confirming completion of abortion (Anger *et al.* 2019). Quantitative point-of-care testing works in a similar way to multilevel pregnancy testing in that a discrete value is given for hCG in serum, urine, or whole blood, and this can be used to compare pre- and post-abortion values to confirm completion of abortion, but unlike a multilevel test, this could not be performed by a patient in their home, due to the use of testing equipment. It is also possible to use high-sensitivity urine pregnancy tests to confirm complete abortion, but usually, this is after a longer time period and there may be a higher rate of false positives than with other methods (Raymond *et al.* 2021).

There are, however, occasions where post-abortion ultrasonography is indicated. Following abortion, if symptoms of pregnancy persist or a low-sensitivity pregnancy test is positive, ultrasonography should be conducted to identify or exclude a viable ongoing pregnancy. However, a common finding is the presence of ultrasonically visible blood and/or pregnancy tissue, which can lead to unnecessary intervention. Retained pregnancy tissue can be challenging to identify and navigate. Ultrasonography cannot infallibly distinguish between a blood clot and adherent decidual tissue. The use of Doppler to identify blood flow to the tissue and correlation with symptoms and physical examination (boggy uterus and open cervix) can give greater certainty regarding the diagnosis (Reynolds-Wright & Fletcher 2021). Even with these additional measures, unnecessary intervention may occur for patients who are universally assessed using post-abortion ultrasonography and in the absence of symptoms.

Where post-abortion ultrasonography is used, cautious interpretation is required when a pre-abortion ultrasonography has not been. An example of this is patients who have not had pre-abortion ultrasonography but have returned to services following medical abortion at home due to pain, bleeding, and positive pregnancy test. Post-abortion ultrasonography may indicate an empty uterus at this stage, but providers need to be alert to the possibility of occult ectopic pregnancy and should consider quantitative hCG monitoring to exclude this.

Post-abortion ultrasonography will also be indicated in cases where abortion has taken place with an IUD *in situ* (Marsh & Cameron 2025) and where expulsion of the IUD had not been visualised at the time of the abortion. Ultrasonography post-abortion in this scenario can confirm whether the IUD is still *in situ* and whether further investigation and intervention to remove it is required.

## Conclusion

Ultrasonography remains an important component of abortion care, as it does in early pregnancy in general. We propose that selective use of ultrasonography, in line with World Health Organization recommendations, allows clinicians and services to maximise the advantages or peri-abortion ultrasound while minimising the impact of the disadvantages of the technology. Patient preferences should be prioritised where possible and safe to do so.

## Declaration of interest

JJRW received research funding from Exelgyn (Nordic Pharma) and HRA Pharma (Perrigo) and speaker fees and meeting attendance support from Gedeon Richter. STC is a member of European advisory board on medical abortion, Exelgyn.

## Funding

This research did not receive any specific grant from any funding agency in the public, commercial, or not-for-profit sector.

## Author contribution statement

JJRW and STC contributed equally to the writing of this invited manuscript.
